# Student Research Involvement as a Supplementary Teaching Approach: A Prospective Evaluation

**DOI:** 10.7759/cureus.91637

**Published:** 2025-09-04

**Authors:** Omar Kiwan, Bisher Tulimat, Adam Buchan, Mohammed Al-Kalbani, Tyler Munro, Ojasvini Chorghade, Omar Abu Baker, Siddharth Kumar, Neha Panyam, Darren Green

**Affiliations:** 1 Faculty of Biology, Medicine and Health, The University of Manchester, Manchester, GBR; 2 School of Medicine, Royal College of Surgeons in Ireland - Bahrain, Busaiteen, BHR; 3 Internal Medicine, Bradford Teaching Hospitals NHS Foundation Trust, Bradford, GBR; 4 Geriatric Medicine, Manchester University NHS Foundation Trust, Manchester, GBR; 5 Anesthesia and Critical Care, Northern Care Alliance NHS Foundation Trust, Salford, GBR; 6 Renal Medicine, Northern Care Alliance NHS Foundation Trust, Salford, GBR

**Keywords:** curriculum development, medical education, medical school, medical students, student research

## Abstract

Introduction

Integrating research into medical education is essential for cultivating evidence-based practitioners and future clinical academics. However, many medical students report limited access to research opportunities, leading to low confidence and competence in conducting research. In addition, most existing student research initiatives are short-term and provide limited continuity and structured teaching. To address this, the Manchester Student Research Network (MSRN) launched an initiative providing long-term research opportunities and structured educational support to students. This work aims to assess how prolonged involvement in research influences students’ perceptions, confidence, and understanding of research.

Methods

A service evaluation was conducted with 66 medical students in the second to final years of training, who participated in a structured research program over nine months. Participation was voluntary, with students applying through the MSRN and selecting only projects they are interested in. Priority was given to clinical-year students with little research experience. The program included diverse research projects, alongside supplementary lectures on research methodology and data analysis. Students completed pre- and post-program surveys to assess their perceptions of research, confidence in research skills, and views on research accessibility. Responses were measured using a Likert scale and binary questions. Statistical analysis included Mann-Whitney U tests for ordinal data and chi-square tests for binary variables, with significance set at p < 0.05.

Results

Confidence significantly improved in eight out of 10 research domains. Median confidence in understanding research methodologies rose from 3 (neutral) to 4 (good) (p < 0.001), reading and analyzing papers from 3 to 4 (p = 0.005), statistical analysis from 2 (poor) to 2.5 (poor - neutral) (p = 0.030), manuscript writing from 3 to 4 (p = 0.012), producing graphs from 2.5 to 3.5 (neutral - good) (p < 0.001), assisting planned research from 3 to 4 (p < 0.001), and leading independent research from 2 to 3 (p = 0.002). Confidence in conducting a literature review also increased significantly without a change in median (p = 0.030). In terms of perceptions and attitudes toward research, the belief that research strengthens portfolios increased from 4 (agree) to 5 (strongly agree) (p = 0.001). Agreement with being “unable to meaningfully contribute to research” fell from 4 to 2 (disagree) (p < 0.001). At the program’s conclusion, 12 students had published their work or were in the process of publishing it, while 37 students reported their project was still ongoing.

Conclusion

Structured, long-term research involvement significantly enhances medical students’ confidence and perceived competence in core research skills. Persistent perceptions of unequal access to research opportunities highlight systemic barriers that must be addressed. Embedding protected research time within medical school curricula, expanding mentorship networks, and creating equitable pathways for research involvement are necessary to foster a more inclusive academic environment. Wider, multi-center studies are needed to validate these findings and establish scalable frameworks for integrating meaningful research experiences into medical education.

## Introduction

Integrating research into undergraduate medical education is essential for developing evidence-based practitioners and inspiring future clinical academics [1]. Research involvement has been shown to enhance students’ understanding of key scientific principles, improve critical thinking skills, and promote long-term engagement with scholarly activities [2,3]. Despite its importance, many medical students report limited access to meaningful research opportunities during their training, leading to low confidence and competence in conducting and understanding research [4,5]. In addition, most existing student research initiatives are short-term and provide limited continuity and structured teaching [6]. 

The Manchester Student Research Network (MSRN) was established in 2024 as a student-led initiative to address these concerns [7]. The network connects medical students with supervisors across multiple specialties, providing a wide range of projects including systematic reviews, clinical research, and lab-based research. Unlike many short-term schemes, the MSRN was designed to offer sustained involvement over an academic year, with structured teaching on research methodology, data analysis, and academic writing. By combining long-term project participation with formal educational support, the MSRN seeks to create an inclusive and scalable model for integrating meaningful research experiences into medical education.

Similar interventions have shown that research involvement enhances students’ research skills and perceptions of academic careers [8,9]. However, much of the current evidence is derived from cross-sectional or retrospective evaluations, which are prone to recall bias and limited by a lack of pre-intervention benchmarking [10,11]. This work aimed to evaluate the impact of the MSRN initiative using prospective pre- and post-intervention questionnaires, assessing changes in students’ perceptions, confidence, and understanding of research after nine months of participation. The authors hypothesized that structured, long-term research involvement would improve medical students’ perceived confidence in research skills, their attitudes toward research, and their understanding of its role in medical education.

## Materials and methods

This study was conducted primarily at The University of Manchester, in partnership with the Northern Care Alliance NHS Foundation Trust, from October 15, 2024, to July 22, 2025. Medical students were followed over a nine-month period during which they engaged in structured research projects. A nine-month timeframe was selected as it aligned with the academic year cycle, ensuring project feasibility within medical training. Students applied to participate through the MSRN, outlining their previous experience in research and academia. Participation was voluntary.

A total of 84 students applied to the program, of which 66 were accepted. Students based in Northwest England were eligible to apply. Selection was based on a scoring system that included year of study, prior research experience, and a scored personal statement outlining motivation for participation. Priority was given to those in clinical years with little or no prior research experience, as they were likely to benefit most from the scheme at their stage of medical training.

Supervisors were recruited through email invitations circulated by regional trusts and academic institutions, as well as online advertisements. Each supervisor completed a standardized form detailing the project and key requirements for students. Students only selected projects they were interested in and were allocated to one of their chosen options. Projects spanned a range of specialties and research types, including systematic reviews and laboratory-based research. Supplementary lectures on research methodology and data analysis were also offered.

A structured survey was conducted both before and after the program, with a nine-month interval between the two surveys. All students involved in the program participated in the survey. As all participants completed both surveys in full, no missing data handling procedures were required. The surveys were designed for the program and were developed in consultation with clinical academics across Northwest England. The surveys were anonymous, and informed consent was obtained electronically in the questionnaire. Both surveys can be found in the Appendices. The survey consisted of 26 questions, asked both before and after research participation, and was divided into three parts: participants' perceptions and attitudes toward research, their confidence in research skills, and their views on research in medical school curricula and specialty applications. Additional questions were included in the initial survey, while the follow-up survey also asked students about the outcome of their research project. The survey used a five-point Likert scale to assess the extent to which participants agreed or disagreed with statements related to their attitudes toward research and their confidence in key research skills. Binary Yes/No questions were used when seeking responses to questions regarding research accessibility and engagement [12].

According to section 3.7 of The University of Manchester's Research Ethics Policy, questionnaires and service evaluations that collect no confidential or sensitive information do not require Institutional Review Board (IRB) review. As the evaluation met the stated criteria, formal IRB approval was not required [13].

Statistical analysis

Data from both surveys were analyzed to compare the significance of changes in response selections before and after the research program. Statistical analysis was conducted using IBM SPSS Statistics for Windows, version 30.0 (Released 2024; IBM Corp., Armonk, New York, United States). A Mann-Whitney U Test was used to determine the significance of the Likert data, and a chi-square test was used for binary Yes/No questions. Statistical significance was set at p < 0.05. Median values were also compared.

## Results

Sixty-six students participated in research projects. Among them, two were in their second year, 34 in their third year, 15 in their fourth year, and 15 in their fifth and final year of training. All students completed both the baseline and follow-up questionnaires and reported active participation in their allocated research project. Of the respondents, 12 students indicated that their research had been published or was in the process of being published. Six students had presented their work at a conference, and two had presented at a local or regional meeting. No project had directly contributed to policy changes, likely due to the limited timescale of the pre- and post-intervention surveys, as influencing policy changes requires a period much longer than nine months. At the time of the follow-up, 37 students reported their research was still ongoing, and 15 students reported that their project had not resulted in publication or presentation and were no longer active. 

Gaining an initial understanding of medical students’ perceptions of research

When asked whether their current medical school curriculum provides sufficient opportunities for research, only 13.6% (9/66) of students agreed or strongly agreed, while 57.6% (38/66) disagreed or strongly disagreed. These findings suggest a prevailing perception that research is inadequately integrated into medical school curricula.

Regarding motivation, 30.3% (20/66) reported a neutral stance toward participating in research due to an interest in a future academic career. Meanwhile, 39.4% (26/66) disagreed or strongly disagreed with this statement, indicating that academic aspirations were not the predominant reason for engaging in research for many participants. 

Perceived barriers to participation were more commonly institutional than personal. Most students (71.2%, 47/66) reported facing systemic barriers within medicine that hindered their ability to participate in research. In contrast, only 42.4% (28/66) reported facing personal barriers, such as limited time, insufficient skills, or a lack of confidence. These results are presented in Table 1.

**Table 1:** Responses to questions in the initial survey to gain an understanding of medical students’ perceptions toward research "–" indicates that data for the respective cell was not obtainable

Statement	I believe current medical school curricula have enough opportunities for research, n (%)	I am partaking in research as I am interested in a career in academia, n (%)	There have been barriers in medicine preventing me from participating in research, n (%)	There have been personal barriers preventing me from participating in research, n (%)
Strongly disagree	9 (13.6%)	9 (13.6%)	–	–
Disagree	29 (43.9%)	17 (25.8%)	–	–
Neutral	19 (28.8%)	20 (30.3%)	–	–
Agree	8 (12.1%)	11 (16.7%)	–	–
Strongly agree	1 (1.5%)	9 (13.6%)	–	–
Median	Disagree	Neutral	–	–
Yes	–	–	47 (71.2%)	19 (28.8%)
No	–	–	28 (42.4%)	38 (57.6%)

Identifying changes in perceptions and attitudes toward research

The results of questions pertaining to perceptions and attitudes of medical students toward research demonstrated a limited shift in medical students’ views. Of the 11 key statements questioned, three saw statistically significant changes in student answers after the nine-month research period. There was a significant decrease in students’ belief that research opportunities should be readily available (p = 0.017), reflecting a negative shift in their perceptions. In contrast, there were two positive changes in students’ views. There was a negative shift in agreement with the statement "I am unable to meaningfully contribute to research initiatives with my current skillset” (p < 0.001), indicating improved confidence in their research abilities, and a positive shift in the belief that research strengthens their portfolios for specialty applications (p = 0.001). Other variables showed no significant change. These results can be found in Table 2. 

**Table 2:** Changes in student perceptions toward research before and after a longitudinal research program

Statement	Research skills are necessary for my future clinical practice	I believe research opportunities should be readily available to students	Learning research methodology is important for my development as a physician	Clinical decisions should be based on sound scientific research	Research is essential for advancing medical knowledge	I am unable to meaningfully contribute to research initiatives with my current skillset	Through research, I can improve my skillset and become a better clinician	Research strengthens my knowledge in a specific and desired specialty	Research allows me to strengthen my portfolio, to better compete	Research negatively impacts my medical studies	I would not partake in research if I was not considering a competitive specialty
Before											
Strongly disagree	1	1	0	0	0	1	0	0	3	9	14
Disagree	1	0	2	0	0	7	1	1	7	19	26
Neutral	7	2	8	3	0	19	2	12	5	23	12
Agree	31	12	36	23	11	28	26	21	29	15	11
Strongly agree	26	51	20	40	55	11	37	22	22	0	3
Median	Agree	Strongly agree	Agree	Strongly agree	Strongly agree	Agree	Strongly agree	Agree	Agree	Neutral	Disagree
After											
Strongly disagree	0	0	1	0	0	5	0	1	0	4	13
Disagree	0	3	2	0	0	34	2	1	0	25	27
Neutral	8	7	6	0	0	8	5	9	1	27	10
Agree	35	20	31	21	12	18	34	36	26	7	10
Strongly agree	23	36	26	45	54	1	25	19	39	3	6
Median	Agree	Strongly agree	Agree	Strongly agree	Strongly agree	Disagree	Agree	Agree	Strongly Agree	Neutral	Disagree
Z-score	0.259	2.380	-0.835	-0.892	0.148	4.930	-1.190	0.196	-3.400	0.575	-0.289
P-value	0.795	0.017	0.401	0.373	0.881	< 0.001	0.234	0.841	0.001	0.569	0.772

Changes in student confidence in research skills

Students rated their confidence in 10 research-related competencies using a five-point Likert scale. Median scores and statistical comparisons are shown in the tables below. Statistically significant improvements were observed in eight out of 10 domains, including understanding of research methodologies (p < 0.001), conducting literature reviews (p < 0.001), reading and analyzing research papers (p < 0.001), statistical analysis (p = 0.030), manuscript writing (p = 0.012), producing graphs (p < 0.001), aiding ongoing research (p < 0.001), and leading independent research projects (p = 0.002). All these domains showed a positive upward trend in students’ confidence pertaining to each question, showing an improvement in their perceived abilities and knowledge of core research skills. Improvements in confidence using data analysis software (p = 0.061) and balancing research with other responsibilities (p = 0.055) trended positively but were not statistically significant. These results can be found in Table 3.

**Table 3:** Self-reported confidence in core research skills before and after participation

Competency	Understanding of research methodologies	Reading and analysing research papers	Conducting a literature review	Statistical analysis	Writing up a paper	Using research software for data analysis	Producing graphs to depict results	Aiding research that has already been planned	Leading and designing your own research project	Balancing your time while partaking in research
Before										
Very poor	3	2	4	15	3	35	10	2	19	1
Poor	18	14	10	28	14	17	23	17	27	8
Neutral	33	27	15	28	29	10	17	23	16	33
Good	12	18	32	5	18	4	12	19	3	22
Very good	0	5	5	0	2	0	4	5	1	2
Median	Neutral	Neutral	Good	Poor	Neutral	Very poor	Poor - Neutral	Neutral	Poor	Neutral
After										
Very poor	1	0	0	7	2	25	0	0	8	0
Poor	6	3	2	26	11	19	11	2	20	5
Neutral	25	28	7	23	15	12	22	23	29	25
Good	31	24	43	8	33	9	30	29	8	29
Very good	3	5	14	2	5	1	3	12	1	7
Median	Good	Good	Good	Poor - Neutral	Good	Poor	Neutral - Good	Good	Neutral	Good
Z-score	-4.05	-2.83	-3.70	-2.11	-2.49	-1.87	-3.84	-3.64	-3.13	-1.92
P-value	< 0.001	0.005	< 0.001	0.030	0.012	0.061	< 0.001	< 0.001	0.002	0.055

Views on research accessibility and engagement

Students were asked a series of binary Yes/No questions, examining their attitudes toward research within the medical curriculum, accessibility of opportunities, and willingness to engage in research beyond core interests.

There was no statistically significant change in students’ belief that research should be a core component of medical education, with high agreement both before (87.9%) and after (83.3%) the program. However, this result could be swayed by the bias of the questioned cohort, being students who voluntarily signed up for the research program. Similarly, students consistently reported low agreement with the idea that research is equally accessible to all medical students, with only 10.6% responding “Yes" at baseline and 4.5% after the intervention. Half of the students agreed post-intervention that research experience should form part of specialty application assessments, compared to 37.9% initially, although this did not represent a statistically significant shift. Students’ comfort with presenting research remained high, with a slight, non-significant decline from 93.9% to 87.9%. Similarly, willingness to engage in research outside of one’s areas of interest remained strong, with a non-significant change from 92.4% before to 89.4% after. These results can be found in Table 4.

**Table 4:** Student views on research accessibility and engagement before and after research participation

Question	Should research be a core component of medical curricula?	Do you believe research is equally accessible to all medical students?	Do you believe research experience should be part of how specialty applicants are assessed?	Would you feel comfortable presenting your research at a conference?	Would you consider getting involved in research outside your areas of interest?
Before					
Yes, n (%)	58 (87.9%)	7 (10.6%)	25 (37.9%)	62 (93.9%)	61 (92.4%)
No, n (%)	8 (12.1%)	59 (89.4%)	41 (62.1%)	4 (6.1%)	5 (7.6%)
After					
Yes, n (%)	55 (83.3%)	3 (4.5%)	33 (50%)	58 (87.9%)	59 (89.4%)
No, n(%)	11 (16.7%)	63 (95.5%)	33 (50%)	8 (12.1%)	7 (10.6%)
Chi-square statistic	0.553	1.730	1.970	1.467	0.367
P-value	0.457	0.188	0.161	0.226	0.545

## Discussion

This evaluation demonstrates that structured, long-term research involvement significantly improves medical students’ perceptions of their research abilities and enhances their confidence in essential research skills. Participation in research led to significant improvements in students’ perceived ability to contribute to research, as well as the belief that research strengthens their portfolios. These findings align with prior literature suggesting that research involvement is effective in nurturing research competencies among medical students [14,15]. Students reported marked improvements in their confidence across multiple core research competencies. Statistically significant improvements were observed in understanding research methodologies, reading and analyzing research papers, conducting literature reviews, writing research papers, visualizing results, statistical analysis, aiding ongoing research, and leading independent research projects. The overall level of confidence remained modest, however, reflecting the fact that these are complex skills and require longer exposure and training. These improvements demonstrate that a combination of practical research experience and supplementary educational support can effectively build research literacy and perceived competence among medical students. 

Students’ perceptions of research accessibility remained largely unchanged. However, there was a significant decline in agreement that research should be readily available to all medical students. Taken together with the significance of research strengthening a candidate's portfolio, this may reflect a belief that students should take personal initiative in seeking out opportunities, especially given the recognized value of research for portfolio development and specialty applications. An alternative explanation is that this decline may reflect disillusionment with systemic barriers or frustration with research accessibility, rather than a genuine shift in attitudes. Nonetheless, the persistence of low agreement regarding research accessibility suggests that while individual initiatives can improve confidence and perceived competence, broader institutional reforms are required to enhance equitable access. Furthermore, the vast majority of participants believed that research is not equally accessible to all medical students, both before and after participation. This perception highlights the need for institutions to identify and address systemic barriers to ensure equitable access to research opportunities. Previous studies have also highlighted institutional limitations, including a lack of attention and cooperation of the medical school’s faculty and administration [16,17]. Structured guidance from faculty and support from senior students may further help sustain engagement and ensure students can effectively navigate the challenges of research participation. Variability in student responses may also reflect differences in individual learning styles and mindsets. Prior work has shown that a positive attitude and mindset encourage students to embrace new challenges and adapt effectively to different tasks, enhancing skill development and academic success [18]. 

The outcomes of students’ research projects reflect the lengthy timelines associated with academic output. While several students had published or presented their work, most projects remained ongoing. This underscores the reality that research dissemination often extends beyond typical academic cycles, highlighting the need for medical school curricula to allocate extended, dedicated time for meaningful research engagement. This is in line with recent evidence showing academic outputs often extend beyond standard academic cycles and that prolonged structured modules can strengthen competencies and sustain engagement [19]. Encouragingly, the sustained engagement of students with their research suggests longitudinal programs may foster continued academic productivity beyond their formal duration.

This analysis is not without limitations. The self-reported nature of the survey introduces potential response and recall bias. The absence of a control group for both the baseline and follow-up questionnaires limits causal inferences regarding the observed changes. The survey also cannot objectively measure competence, weakening the reliability of the results. The nine-month follow-up period may not be sufficient to capture long-term research outcomes, such as eventual publication or contribution to policy changes. Statistical analysis did not adjust for multiple comparisons with the large number of tests performed. In addition, the cohort consisted of students who voluntarily enrolled in the MSRN program, suggesting a pre-existing interest in research. This self-selection may limit the generalizability of the findings and could introduce bias, as their responses may be more positive than those of the broader student population. While the questionnaires were developed with input from clinical academics, they were not formally validated. This may limit the reliability and construct validity of the findings. Another limitation is that confidence intervals and conventional effect sizes were not reported. As the analysis relied on non-parametric methods for data, these measures are less commonly applied in this context. Nonetheless, their absence may limit the ability to interpret the magnitude of change beyond statistical significance. No formal power calculation was performed as this was an exploratory service evaluation. While all eligible applicants were included to maximize sample size, the absence of a priori sample size estimation may limit the strength of statistical inferences. The modest sample size precluded meaningful subgroup analysis. Furthermore, the cohort evaluated also consisted solely of medical students from the Northwest of England, which may limit the applicability of these findings. Future research involving students across multiple regions will be essential to validate these results and assess the broader effects of student research involvement. 

## Conclusions

This evaluation demonstrates that structured, long-term research involvement significantly enhances medical students’ confidence and perceived competence in key research skills. Students reported notable improvements in their ability to understand research methodologies, critically appraise literature, conduct statistical analyses, and meaningfully contribute to research projects. These findings underscore the importance of long-term research involvement, combined with structured teaching, in bridging the gap between theoretical research knowledge and practical competence among medical students.

Persistent perceptions of unequal research accessibility highlight ongoing systemic barriers that must be addressed. Integrating protected research time within medical school curricula, expanding mentorship networks, and creating equitable pathways for research involvement are important steps forward. As this was a single-center, self-selected cohort with self-reported outcomes, the findings should be interpreted cautiously. Larger, multi-center studies with methodological improvements are needed to build on these findings and to establish scalable models for embedding worthwhile research experiences in medical education.

## Appendices

Survey questionnaires 

**Table 5:** Pre-participation questionnaire

Initial student questionnaire					
Section 1: Please indicate the extent to which you agree or disagree with the following statements					
I believe current medical school curricula have enough opportunities for research					
Strongly Disagree	Disagree	Neutral		Agree	Strongly agree
I am partaking in research as I am interested in a career in academia					
Strongly Disagree	Disagree	Neutral		Agree	Strongly agree
There have been barriers in medicine preventing me from participating in research					
Yes			No		
There have been personal barriers preventing me from participating in research					
Yes			No		
Research skills are necessary for my future clinical practice					
Strongly Disagree	Disagree	Neutral		Agree	Strongly agree
I believe research opportunities should be readily available to students					
Strongly Disagree	Disagree	Neutral		Agree	Strongly agree
Learning research methodology is important for my development as a physician					
Strongly Disagree	Disagree	Neutral		Agree	Strongly agree
Clinical decisions should be based on sound scientific research					
Strongly Disagree	Disagree	Neutral		Agree	Strongly agree
Research is essential for advancing medical knowledge					
Strongly Disagree	Disagree	Neutral		Agree	Strongly agree
I am unable to meaningfully contribute to research initiatives with my current skillset					
Strongly Disagree	Disagree	Neutral		Agree	Strongly agree
Through research, I can improve my skillset and become a better clinician					
Strongly Disagree	Disagree	Neutral		Agree	Strongly agree
Research strengthens my knowledge in a specific and desired specialty					
Strongly Disagree	Disagree	Neutral		Agree	Strongly agree
Research allows me to strengthen my portfolio, to better compete					
Strongly Disagree	Disagree	Neutral		Agree	Strongly agree
Research negatively impacts my medical studies					
Strongly Disagree	Disagree	Neutral		Agree	Strongly agree
I would not partake in research if I was not considering a competitive specialty					
Strongly Disagree	Disagree	Neutral		Agree	Strongly agree
Section 2: Please rate your confidence in the following skills					
Understanding of research methodologies					
Very Poor	Poor	Neutral		Good	Very Good
Reading and analysing research papers					
Very Poor	Poor	Neutral		Good	Very Good
Conducting a literature review					
Very Poor	Poor	Neutral		Good	Very Good
Statistical analysis					
Very Poor	Poor	Neutral		Good	Very Good
Writing up a paper					
Very Poor	Poor	Neutral		Good	Very Good
Using research software for data analysis					
Very Poor	Poor	Neutral		Good	Very Good
Producing graphs to depict results					
Very Poor	Poor	Neutral		Good	Very Good
Aiding research that has already been planned					
Very Poor	Poor	Neutral		Good	Very Good
Leading and designing your own research project					
Very Poor	Poor	Neutral		Good	Very Good
Balancing your time while partaking in research					
Very Poor	Poor	Neutral		Good	Very Good
Section 3: Please let us know your answer to the following questions					
Should research be a core component of medical curricula?					
Strongly Disagree			Disagree		
Do you believe research is equally accessible to all medical students?					
Yes			No		
Do you believe research experience should be part of how specialty applicants are assessed?					
Yes			No		
Would you feel comfortable presenting your research at a conference?					
Yes			No		
Would you consider getting involved in research outside your areas of interest?					
Yes			No		

**Table 6:** Post-participation questionnaire

Follow-up student questionnaire					
Section 1: Please indicate the extent to which you agree or disagree with the following statements					
Research skills are necessary for my future clinical practice					
Strongly Disagree	Disagree	Neutral		Agree	Strongly agree
I believe research opportunities should be readily available to students					
Strongly Disagree	Disagree	Neutral		Agree	Strongly agree
Learning research methodology is important for my development as a physician					
Strongly Disagree	Disagree	Neutral		Agree	Strongly agree
Clinical decisions should be based on sound scientific research					
Strongly Disagree	Disagree	Neutral		Agree	Strongly agree
Research is essential for advancing medical knowledge					
Strongly Disagree	Disagree	Neutral		Agree	Strongly agree
I am unable to meaningfully contribute to research initiatives with my current skillset					
Strongly Disagree	Disagree	Neutral		Agree	Strongly agree
Through research, I can improve my skillset and become a better clinician					
Strongly Disagree	Disagree	Neutral		Agree	Strongly agree
Research strengthens my knowledge in a specific and desired specialty					
Strongly Disagree	Disagree	Neutral		Agree	Strongly agree
Research allows me to strengthen my portfolio, to better compete					
Strongly Disagree	Disagree	Neutral		Agree	Strongly agree
Research negatively impacts my medical studies					
Strongly Disagree	Disagree	Neutral		Agree	Strongly agree
I would not partake in research if I was not considering a competitive specialty					
Strongly Disagree	Disagree	Neutral		Agree	Strongly agree
Section 2: Please rate your confidence in the following skills					
Understanding of research methodologies					
Very Poor	Poor	Neutral		Good	Very Good
Reading and analysing research papers					
Very Poor	Poor	Neutral		Good	Very Good
Conducting a literature review					
Very Poor	Poor	Neutral		Good	Very Good
Statistical analysis					
Very Poor	Poor	Neutral		Good	Very Good
Writing up a paper					
Very Poor	Poor	Neutral		Good	Very Good
Using research software for data analysis					
Very Poor	Poor	Neutral		Good	Very Good
Producing graphs to depict results					
Very Poor	Poor	Neutral		Good	Very Good
Aiding research that has already been planned					
Very Poor	Poor	Neutral		Good	Very Good
Leading and designing your own research project					
Very Poor	Poor	Neutral		Good	Very Good
Balancing your time while partaking in research					
Very Poor	Poor	Neutral		Good	Very Good
Section 3: Please let us know your answer to the following questions					
Should research be a core component of medical curricula?					
Yes			No		
Do you believe research is equally accessible to all medical students?					
Yes			No		
Do you believe research experience should be part of how specialty applicants are assessed?					
Yes			No		
Would you feel comfortable presenting your research at a conference?					
Yes			No		
Would you consider getting involved in research outside your areas of interest?					
Yes			No		
Section 4: Please let us know the outcome of your project					
Project outcome					
Published or in the process of publishing	Presented at a conference	Presented at a local or regional meeting		Directly contributed to policy changes	None of the above
